# ConvNeXt-Driven Detection of Alzheimer’s Disease: A Benchmark Study on Expert-Annotated AlzaSet MRI Dataset Across Anatomical Planes

**DOI:** 10.3390/diagnostics15232997

**Published:** 2025-11-25

**Authors:** Mahdiyeh Basereh, Matthew Alexander Abikenari, Sina Sadeghzadeh, Trae Dunn, René Freichel, Prabha Siddarth, Dara Ghahremani, Helen Lavretsky, Vivek P. Buch

**Affiliations:** 1 Department of Computer Science, Allameh Tabataba’i University, Tehran 1489684511, Iran; mahdiyeh_basereh@atu.ac.ir; 2 Department of Neurosurgery, Stanford University School of Medicine, Stanford, CA 94305, USA; mattabi@stanford.edu (M.A.A.); sinas@stanford.edu (S.S.); traedunn@stanford.edu (T.D.); 3 Department of Psychology, University of Amsterdam, 1018 WS Amsterdam, The Netherlands; r.freichel@uva.nl; 4 Department of Psychiatry and Biobehavioral Sciences and the Semel Institute for Neuroscience, University of California, Los Angeles, CA 90095, USA; psiddarth@mednet.ucla.edu (P.S.); darag@ucla.edu (D.G.); hlavretsky@mednet.ucla.edu (H.L.)

**Keywords:** Alzheimer’s disease, ConvNeXt, deep learning, structural MRI, coronal plane, computer-aided diagnosis, transfer learning, neuroimaging biomarkers

## Abstract

**Background:** Alzheimer’s disease (AD) is a leading worldwide cause of cognitive impairment, necessitating accurate, inexpensive diagnostic tools to enable early recognition. **Methods:** In this study, we present a robust deep learning approach for AD classification based on structural MRI scans, ConvNeXt, an emergent convolutional architecture inspired by vision transformers. We introduce AlzaSet, a clinically curated T1-weighted MRI dataset of 79 subjects (63 with Alzheimer’s disease [AD], 16 cognitively normal controls [NC]) acquired on a 1.5 T Siemens Aera in axial, coronal, and sagittal planes, respectively (12,947 slices in total). Images are neuroradiologist-labeled. Results are reported per plane, with awareness of the class imbalance at the subject level. We further present AlzaSet, a novel, expertly labeled clinical dataset with axial, coronal, and sagittal perspectives from AD and cognitively normal control subjects. Three ConvNeXt sizes (Tiny, Small, Base) were compared and benchmarked against existing state-of-the-art CNN models (VGG16, VGG19, InceptionV3, DenseNet121). **Results:** ConvNeXt-Base consistently outperformed the other models on coronal slices with an accuracy of 98.37% and an AUC of 0.992. Coronal views were determined to be most diagnostically informative, with emphasis on visualization of the medial temporal lobe. Moreover, comparison with recent ensemble-based techniques showed superior performance with comparable computational efficiency. **Conclusions:** These results indicate that ConvNeXt-capable models applied to clinically curated datasets have strong potential to provide scalable, real-time AD screening in diverse settings, including both high-resource and resource-constrained settings.

## 1. Introduction

Dementia is a rapidly growing global health concern, affecting an estimated 57 million individuals worldwide, with approximately 10 million new cases reported each year. As the seventh leading cause of mortality across all age groups, it represents a significant and rising public health burden [1]. Alzheimer’s disease (AD) accounts for 60–70% of all dementias and is associated with a significant burden on both patients and caregivers, as well as health care systems and society in general. Addressing AD has become an imperative given the increase in individuals aged over 60 [2]. This demographic pattern calls for preventive healthcare planning and the creation of faster and more accurate diagnostic tools, deployable at scale [3].

Beyond clinical and biological determinants, recent work highlights that effective health interventions, particularly for vulnerable or stigmatized populations, depend on how actors navigate complex stakeholder values and local social dynamics. Studies of restorative entrepreneurship demonstrate that sustained, value-aligned engagement with marginalized groups can mitigate exclusion, improve access to care, and enable community-driven solutions, even in resource-constrained or high-stigma environments [4]. These insights underscore the broader need for diagnostic and care strategies in AD that account not only for biological markers but also for the social infrastructures shaping patients’ access, participation, and outcomes.

AD is a chronic, progressive neurodegenerative disorder marked by structural and functional impairment of the brain, predominantly in the hippocampus and associated cortical regions. The hippocampus, which is a critical structure for learning and memory, is often among the first and most severely affected regions in AD. Hippocampal atrophy, which can be identified by structural magnetic resonance imaging (MRI), is highly correlated with the severity of disease and is an acceptable imaging biomarker [5].

Recent work has also explored the value of peripheral and molecular biomarkers in parallel with imaging, though their clinical translation remains limited. Elevated levels of plasma Aβ40 and Aβ42, for example, have been linked to subjective memory changes in older adults, raising the possibility that peripheral amyloid burden can serve as an early signal of cognitive vulnerability [6]. Similarly, integrative approaches in other neurological and psychiatric conditions, such as trigeminal neuralgia and depression, have shown that combining imaging features with immune and inflammatory markers can improve disease subtyping and prognosis [7,8]. Collectively, these findings highlight the promise of multimodal biomarker strategies. However, compared to invasive or costly measures such as tau PET or plasma assays, structural MRI remains the most widely accessible and clinically scalable modality. Importantly, MRI directly captures the core neurobiological hallmark of AD, the progressive atrophy of the hippocampus and medial temporal lobe. Coronal MRI slices provide optimal visualization of these structures, enabling automated models to learn biologically relevant features.

Recent studies have also underscored the growing focus on multimodal and explainable AI approaches for Alzheimer’s diagnosis. A comprehensive review of explainable AI methods emphasized that while deep learning models achieve high accuracy, their limited interpretability remains a major barrier to clinical adoption [9]. Subsequent work demonstrated that combining MRI with clinical features through meta-learning strategies can improve both predictive performance and transparency of model decision-making [10]. Other approaches have further incorporated rule-based logic and visualization modules to enhance interpretability and clinician trust in diagnostic predictions [11]. Together, these findings highlight a growing movement toward multimodal, interpretable frameworks.

Over the past decade, deep learning (DL) has revolutionized medical image analysis through the delivery of automated, scalable, and highly accurate solutions to complex classification tasks. DL is a subcategory of machine learning that utilizes multi-layered artificial neural networks capable of learning hierarchical features from data, often without manual preparation of inputs. In computer vision and medical imaging, DL-based models have consistently outperformed traditional rule-based and shallow learning approaches [12]. Despite this progress, one of the biggest challenges in applying DL to medicine is the scarcity of annotated data, stemming from privacy restrictions and the intensive resources required for expert labeling. As a result, many prior studies in AD neuroimaging have relied on small datasets and insufficient validation procedures. Systematic reviews have highlighted that these limitations can lead to inflated performance estimates due to issues such as data leakage or overfitting [13]. Addressing these concerns requires rigorously curated datasets and carefully designed training pipelines that directly link model performance to disease-relevant biology. Transfer learning has emerged as an effective method to mitigate data scarcity by leveraging pre-trained models trained on large datasets such as ImageNet [14].

Convolutional Neural Networks (CNNs), the most commonly used DL models for image processing, represent images in a hierarchical manner with increasing abstraction through their hierarchy. Early layers extract low-level features like edges, gradients, and textures while deeper layers capture higher-order semantic features of objects. Although pre-trained on natural images, CNNs used for image processing demonstrate phenomenal cross-domain adaptability; their hierarchies can readily be fine-tuned to medical imaging domains, hence enabling faster convergence and reducing overfitting on small datasets [15,16].

Contemporary advances in CNN structures like AlexNet, VGG, ResNet, and Inception have attained remarkable performance on many visual recognition tasks. Most recently, ConvNeXt, a model created by researchers at Facebook AI Research (FAIR) in 2022, set new records for convolutional network performance. ConvNeXt is an enhanced ConvNet architecture inspired by Vision Transformer (ViT) design principles. By combining architectural innovation and updated training methods, ConvNeXt achieves best-in-class performance on a variety of computer vision tasks, surpassing performance of both transformer-based and CNN models with computational efficiency [17]. In the context of AD, ConvNeXt is particularly well-suited for detecting subtle hippocampal and medial temporal lobe atrophy on MRI, the primary hallmark of AD progression.

Beyond binary AD/NC classification, several works approach multi-class staging, considering for example, AD/MCI/NC or AD/EMCI/LMCI/NC classes using hybrid pipelines combining feature selection strategies with CNNs. Representative examples include mRMR-guided feature selection combined with CNN classifiers, stacking/ensemble models, and attention-augmented ConvNeXt/ResNet variants on public datasets. In particular, Eroglu et al. demonstrated classification of Alzheimer’s disease severity on brain MRI using a 4-class public Kaggle set (Non-Demented, Very-Mild, Mild, Moderate) [18]. They extract features with three pre-trained CNNs (Darknet53, InceptionV3, ResNet101), concatenate them, apply mRMR feature selection, and train shallow classifiers (KNN, SVM). The hybrid feature-fusion + KNN model reports 99.1% overall accuracy-outperforming each backbone alone-on five-fold evaluation in MATLAB (R2024a and R2024b) without image preprocessing. Strong points are the simple, model-agnostic fusion pipeline and strong headline metrics; the main weaknesses are reliance on a nonclinical Kaggle dataset with marked class imbalance (very few “Moderate” cases), image-level rather than subject-level splitting (with potential leakage), lack of external/clinical validation, and missing patient metadata-so that generalizability to real clinical cohorts remains unproven. These studies point out the promise and the complexity of four-class paradigms while at the same time underlining the requirement for clinically curated datasets and transparent benchmarking against simpler, deployment-ready architectures.

In this paper, we present a ConvNeXt-inspired design for AD detection from structural MRI scans. To encourage and enable reproducibility in the field, we introduce AlzaSet, a new expert-annotated dataset of AD patients and healthy, cognitively normal controls. Using this approach, we aimed to provide more accurate and accessible diagnostic support tools for clinicians and radiologists, with a powerful tool in hand to diagnose AD at an earlier stage and reduce rates of underdiagnosis in the clinic.

Deep convolutional neural networks (CNNs) are now normative in medical image analysis, particularly in neuroimaging applications such as Alzheimer’s disease (AD) detection. Their hierarchical structure entails automatic feature learning from imaging data, with deeper layers containing more abstract representations. Transfer learning from large-scale pretrained models has in recent years become a feasible solution for addressing the limitations due to small medical datasets and making successful model generalization with limited data possible.

CNNs originally developed for classifying natural images have been found to be successfully adapted for neuroimaging with transfer learning. Liu et al. [17,19] employed a pre-trained model of AlexNet to differentiate MR images into pathological or non-pathological and reported 100% accuracy using a Harvard Medical School dataset (*n* = 215).

Other researchers have implemented comparative assessment across a range of pretrained architectures. A comparison of AlexNet, GoogleNet, and ResNet101 on classification of ADNI data indicated that both AlexNet and GoogleNet achieved area under the curve (AUC) results greater than 89%, and demonstrated clear superiority over CNNs without transfer learning or pretraining [20]. Mehmood et al. [21] obtained 98.73% classification accuracy by employing a variant of VGG19 by freezing different convolutional blocks and using data augmentation to address class imbalance. Similarly, in [22], the authors utilized a stepwise fine-tuning and freezing method with VGG16 and VGG19, further improving performance with manually pre-designed fully connected layers for dementia stage classification. Their models obtained 96.39% and 96.81% accuracy, respectively, and outperformed DenseNet and ResNet50 for the same task.

Ensemble approaches have also been explored to enhance predictive capacity. For instance, Ref. [23] proposed two ensemble architectures: one combining VGG19 and Xception (achieving 93% accuracy), and another that combined ResNet50 and VGG16 (achieving 95%). In [24], various models (VGG16, VGG19, AlexNet) were compared with baseline machine learning classifiers support vector machines (SVM) and random forest (RF). The deep learning models, particularly those that used transfer learning, significantly outperformed traditional methods, with accuracies of 98–100% as opposed to 81.6% (SVM) and 85% (RF).

Later works concentrated on employing more recent CNN model architectures such as ConvNeXt for the detection of AD. ConvNeXt was integrated with a pipeline of 3D convolution and 3D Squeeze-and-Excitation (SE) attention in [25] and attained an accuracy of 94.23% for AD vs. normal controls. Another study [26] employed ConvNeXt on the ADNI database to make predictions about AD stages and compared its performance against ResNet50 and Swin Transformer, a high-performance ConvNeXt outperformed the two alternatives with 95.3% accuracy compared to 93.8% (ResNet50) and 94.3% (Swin Transformer), which shows that novel CNN architectures can achieve superior or equivalent performance to transformers in medical image classification tasks with limited training data.

Collectively, these studies demonstrate the ability of transfer learning and modern CNN architectures to predict AD based on neuroimaging. However, the majority of such studies rely on high-dimensional 3D imaging or complex ensemble pipelines that are usually computationally intensive and less convenient for implementation in the clinical setting. On the other hand, 2D slice-based models, particularly those learned across multiple anatomical planes, offer significant advantages in computational cost, scalability, and compatibility with existing imaging infrastructures. While they are straightforward, fewer efforts have comprehensively optimized 2D models like ConvNeXt with domain-specific preprocessing and benchmarking performance along the orientation axis. Thus, the purpose of this study is to demonstrate the effectiveness of an optimized 2D ConvNeXt framework trained on the AlzaSet dataset for accurate, efficient, and clinically scalable Alzheimer’s disease classification.

This work investigates whether a streamlined, end-to-end ConvNeXt framework trained on a clinically curated dataset can achieve state-of-the-art MRI-based AD screening performance without ensembles or attention-heavy 3D extensions. Our contributions are as follows: (i) AlzaSet: an expert-labeled, clinically acquired T1 dataset with standardized acquisition across three planes; (ii) an orientation-aware analysis demonstrating coronal-slice superiority that is concordant with medial temporal lobe neurobiology; (iii) a ConvNeXt-Base design that achieves 98.37% accuracy (AUC = 0.992) on coronal images with an efficient 2D pipeline; (iv) a dedicated comparison against recent ConvNeXt-based benchmarks to detail incremental advances; and (v) a candid limitations analysis (age and class imbalance, single-site data, binary endpoint) with a roadmap for longitudinal, multimodal, and multi-site validation.

## 2. Materials and Methods

### 2.1. Dataset Description: The AlzaSet Cohort

This study introduces a novel, expertly annotated dataset termed AlzaSet, comprising whole-brain structural T1-weighted MRI scans from individuals diagnosed with Alzheimer’s disease (AD) and cognitively normal control (NC) subjects. The dataset includes T1-weighted MRI scans from a total of 79 participants, 63 with clinically diagnosed AD and 16 cognitively normal controls. Data collection was conducted in collaboration with the Radiology Department at Sasan Hospital (Tehran, Iran), and all images were reviewed and labeled by experienced neuroradiologists. Imaging was performed using a Siemens MAGNETOM Aera 1.5 Tesla MRI scanner (Siemens Healthineers, Erlangen, Germany), equipped with Tim^®^ 4G integrated coil technology and Dot™ (Day optimizing throughput) workflow protocols. Each MRI session included standard neuroanatomical orientations, axial, coronal, and sagittal planes, to ensure comprehensive spatial coverage of the brain. Demographic information of the participants is presented in Table 1.

To facilitate image processing, each frame of the scanned images was converted from DICOM (.dcm) to JPEG (.jpg) format while preserving original dimensions and resolution. See Table 2 for complete numerical distributions.

The dataset was split into training, validation and test subsets to optimize classification algorithm performance. The details are presented in Table 3.

### 2.2. Data Preprocessing and Augmentation Strategy

**Figure 1:**
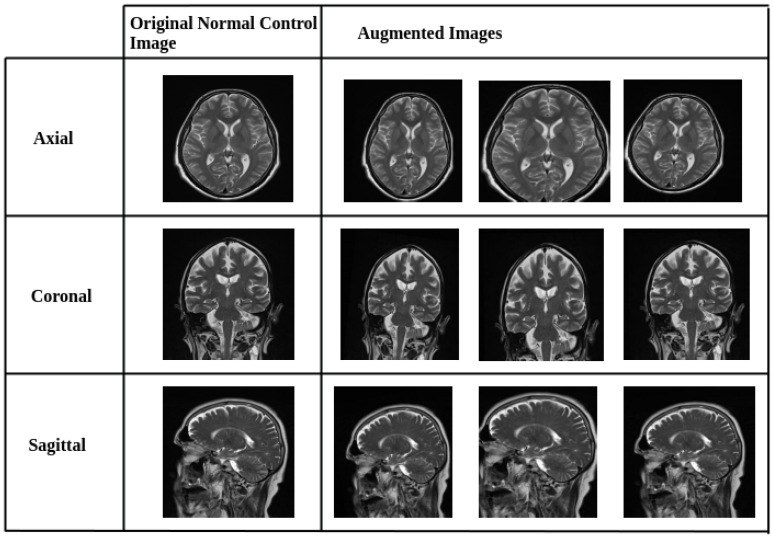
Representative Data Augmentation Pipeline for Normal Control (NC) Class. Sample images from the NC cohort after applying geometric data augmentation techniques. Transformations include horizontal and vertical shifts (±5%), 5-degree rotations, and 0.05× zoom scaling. These augmentations were applied to address class imbalance by synthetically expanding the NC dataset, thereby improving model generalization and reducing overfitting.

To mitigate class imbalance between Alzheimer’s disease (AD) and normal control (NC) images in the AlzaSet dataset, we applied partial data augmentation exclusively to the underrepresented NC cohort. Geometric transformations, including 5% horizontal and vertical translations, 5° rotations, and 0.05× zoom scaling, were employed to synthetically expand the NC subset and approximate numerical parity with AD cases. Representative augmented images from the NC group are illustrated in Figure 1.

Following augmentation, all images were resized to a standardized input dimension of 224 × 224 pixels. Pixel intensities were normalized from the original [0, 255] grayscale range to a floating-point range of [0, 1] to improve numerical stability and reduce computational complexity during model training. The final post-augmentation distribution of the dataset is summarized in Table 4.

### 2.3. Model Architecture and Implementation of Transfer Learning and Feature Extraction Strategy Using ConvNeXt Architecture

Convolutional neural networks (CNNs) extract higher-level abstract features with hierarchical layers. The initial convolutional layers tend to learn the low-level features such as edges, texture, and gradients that have high domain-transferability even in training on natural image datasets. For medical imaging, transfer learning using feature extraction is a good way of avoiding overfitting as well as enhancing generalizability in the case of limited amounts of data available. With the pretrained convolutional layers frozen, the models retain representations that are generally applicable but fine-tune the classification layers with task-specific data [27].

**Figure 2:**
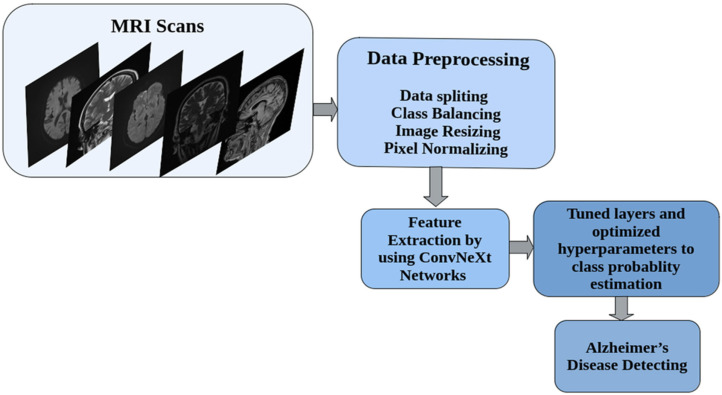
End-to-End Workflow for MRI-Based Alzheimer’s Disease Classification Using ConvNeXt. Overview of the full pipeline for classifying Alzheimer’s disease from structural MRI data. Raw MRI scans in axial, coronal, and sagittal planes undergo a preprocessing stage involving data splitting, class balancing through augmentation, image resizing, and pixel normalization. Preprocessed images are passed through frozen convolutional blocks of ConvNeXt networks for feature extraction. The extracted features are then processed by a set of task-specific layers with optimized hyperparameters to generate final class probability estimates for Alzheimer’s disease diagnosis.

We utilized the first three blocks of the ConvNeXt model as a pre-trained fixed feature extractor in this work. The pre-trained weights on ImageNet were not changed while the remaining layers were tuned and optimized for AD classification. As observed from Figure 2, the extracted feature maps were input to a customized classification head trained to identify neurodegenerative patterns within structural MRI scans.

### 2.4. ConvNeXt-Based Model Design Architecture

ConvNeXt is a multi-stage convolutional neural network that integrates design principles from ResNet and Swin Transformer architectures, but is built entirely with convolutional operations. It comprises four consecutive stages and incorporates substantial improvements, larger convolutional kernels (7 × 7), depthwise separable convolutions, and residual connections, which boost its receptive field while maintaining computational efficiency [17]. ConvNeXt employs non-overlapping convolutional blocks with stride-adjusted kernels, and its core architectural building blocks are revamped residual blocks that adopt Transformer-inspired improvements without sacrificing the simplicity of conventional ConvNeXts.

In this study, we developed modified ConvNeXt-Tiny, ConvNeXt-Small, and ConvNeXt-Base architectures, using the original pretrained models as backbones and adding custom adaptations for medical imaging classification:

- **ConvNeXt-Tiny** consists of four stages with [3, 3, 9, 3] blocks, respectively. We preserved the first 125 layers and truncated the final 25 layers to prevent overfitting and reduce model complexity. Each block implements the sequence: **Input → LayerNorm → PWConv (1 × 1) → GELU → DWConv (7 × 7) → PWConv (1 × 1) → Residual Add**, where **DWConv** denotes depthwise convolution and **PWConv** denotes pointwise convolution.
- **ConvNeXt-Small** shares the same block structure as Tiny but incorporates deeper feature extraction in Stage 3, with [3, 3, 27, 3] blocks. We retained the first 240 layers and removed the final 50 layers for this configuration. Furthermore, the deeper feature extraction results from the iterative application of blocks rather than being an inherent component of the architecture itself.
- **ConvNeXt-Base** expands upon the Small variant by increasing channel widths for greater representational capacity. We extracted features from the first 245 layers, truncating the final 45 layers.

**Figure 3:**
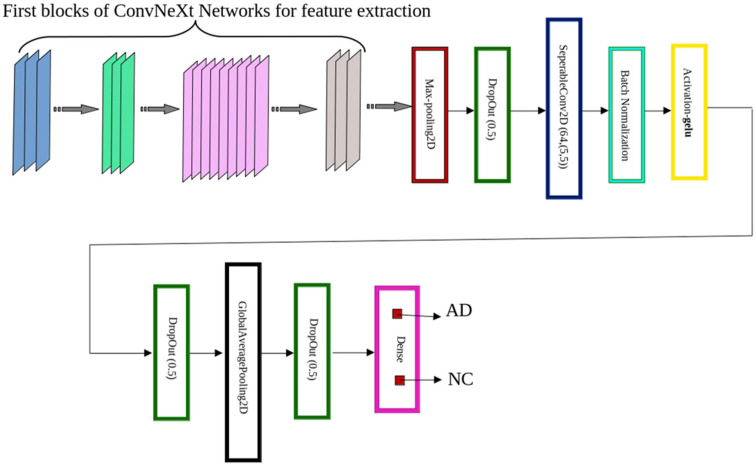
Architecture of the Proposed ConvNeXt-Based Alzheimer’s Disease Classifier. Schematic representation of the model architecture used for Alzheimer’s disease detection. The initial layers of a pretrained ConvNeXt variant (Tiny, Small, or Base) are used for feature extraction. These are followed by a custom classification head comprising a MaxPooling2D layer, Dropout (rate = 0.5), SeparableConv2D, Batch Normalization, and GELU activation. The output is further processed by GlobalAveragePooling2D, additional Dropout layers, and a final Dense layer with two output neurons activated by softmax to classify inputs as either Alzheimer’s disease (AD) or normal control (NC).

As illustrated in Figure 3, each ConvNeXt-based backbone generates a high-dimensional feature map, which is subsequently passed through a custom classification head consisting of the following components:

1. **MaxPooling2D**—A spatial pooling operation (e.g., 2 × 2) that downsamples the feature map by selecting the maximum activation within each region. This reduces dimensionality, accelerates computation, and mitigates overfitting by emphasizing salient features [28].
2. **Dropout (rate = 0.5)**—A regularization technique that randomly deactivates 50% of neurons during training, improving generalization by preventing co-adaptation of feature detectors [29].
3. **SeparableConv2D**—Implements a two-step process: (i) **depth-wise convolution**, which applies a spatial filter independently to each input channel, followed by (ii) **pointwise convolution** (1 × 1) to mix inter-channel information. This architecture offers significant computational savings while retaining competitive performance [30].
4. **Batch Normalization**—Normalizes each feature map by applying consistent mean and variance across spatial dimensions. This stabilizes and accelerates training, especially in deep networks [31].
5. **GELU Activation**—The Gaussian Error Linear Unit (GELU) activation, defined as:
  (1) $$GELU(x)=x\Phi (x)=x\cdot \frac{1}{2}[1+erf(x/\sqrt{2})]$$
  where Φ(x) represents the standard Gaussian cumulative distribution function and erf denotes the error function. The GELU outperforms other activation functions such as ReLU and ELU across multiple tasks, as demonstrated in the experiments [32].
6. **GlobalAveragePooling2D**—Reduces each feature map to a single scalar by averaging across all spatial dimensions (height and width), effectively summarizing global context while minimizing parameter count [33].
7. **Dense Layer with Softmax Activation**—A fully connected output layer containing two neurons, representing the binary classes (AD and NC). The softmax activation function generates normalized probability scores for final classification.

### 2.5. Training Configuration and Hyperparameters

To optimize model performance, we carefully selected hyperparameters based on existing literature in the field of deep learning and task-specific requirements [17,18,19]. Table 5 summarizes the key training hyperparameters and their configurations.

Performance Evaluation

Classification Metrics

To validate the correctness of a classification task, we compute four measures:

1. **True Positives (tp):** Correctly identified instances of the class.
2. **True Negatives (tn):** Correctly rejected instances that do not belong to the class.
3. **False Positives (fp):** Instances wrongly classified as part of the class.
4. **False Negatives (fn):** Actual class instances that were missed. These measurements contribute **Confusion Matrix** that presented in Table 6 [34].

We evaluate our proposed model performance using standard metrics: Accuracy, Precision, Recall (Sensitivity), F1-Score and AUC, which are computed based on the confusion matrix.

Accuracy, computed as the proportion of correctly predicted instances relative to the total predictions, represents a fundamental metric for evaluating model efficiency in classification tasks. Accuracy is obtained from Equation (2) [35].

(2) $$Accuracy=\frac{tp+tn}{tp+fn+fp+tn}$$

Precision, calculated using Equation (3), represents the proportion of correctly predicted positive instances among all positive predictions (i.e., cases identified as AD in our study) [36].

(3) $$Precision=\frac{tp}{tp+fp}$$

Recall (Sensitivity) is computed as the proportion of correct AD cases predicted among all actual AD cases (Equation (4))

(4) $$Recall=\frac{tp}{tp+fn}$$

F1-Score (Equation (5)) provides the harmonic mean of Precision and Recall.

(5) $$F1-Score=\frac{2\times (Precision\times Recall)}{\left(Precision+Recall\right)}$$

The Area Under the ROC Curve (AUC) is a scalar metric that measures the model’s ability to discriminate between positive (AD) and negative (NC) classes [37].

## 3. Results

### 3.1. Model Performance Across Anatomical Planes

This section presents a comprehensive analysis of our proposed architecture’s performance across multiple ConvNeXt configurations (Tiny, Small, and Base variants) using the aforementioned evaluation metrics. The benchmark results are obtained on our newly introduced AlzaSet Dataset. Classification results across anatomical planes are presented as follows:

Table 7 (Axial Plane): Diagnostic accuracy and evaluation metrics for transverse view analysis.

Table 8 (Coronal Plane): Diagnostic accuracy and evaluation metrics for frontal view analysis.

Table 9 (Sagittal Plane): Diagnostic accuracy and evaluation metrics for lateral view analysis. Table 10, Table 11 and Table 12 also demonstrate the performance of pre-trained networks on Axial, Coronal, and Sagittal plane images of AlzaSet.

We compared the performance of three versions of ConvNeXt, Tiny, Small, and Base, on the AlzaSet dataset in terms of classification performance across axial, coronal, and sagittal anatomical views. Overall, with all views taken together, the ConvNeXt-Base model performed better than its smaller counterparts and had the best values of accuracy, precision, recall, F1-score, and AUC. For the axial plane, ConvNeXt-Base scored 92.08% accuracy and an AUC of 0.829, lagging behind ConvNeXt-Tiny slightly in accuracy but showing better balance between precision and recall. ConvNeXt-Small demonstrated the weakest performance among the three variants, likely due to its limited depth and a suboptimal balance between model complexity and overfitting risk.

Sagittal plane performance also maintained a similar trend, where ConvNeXt-Base achieved a 92.09% classification accuracy and an AUC of 0.779. Tiny trailed closely behind, followed by the Small variant with lower classification accuracy. ConvNeXt-Base delivered its highest performance in the coronal plane, achieving a 98.37% classification accuracy and 0.992 AUC. ConvNeXt-Tiny also delivered an excellent performance in this view, achieving a close to 99% accuracy. These findings are a validation set for diagnostic utility of coronal images, which provide superior anatomical definition of medial temporal lobe structures engaged early in AD pathology. They include the hippocampus and the parahippocampal gyrus.

### 3.2. Comparative Benchmarking Against Established Models

**Figure 4:**
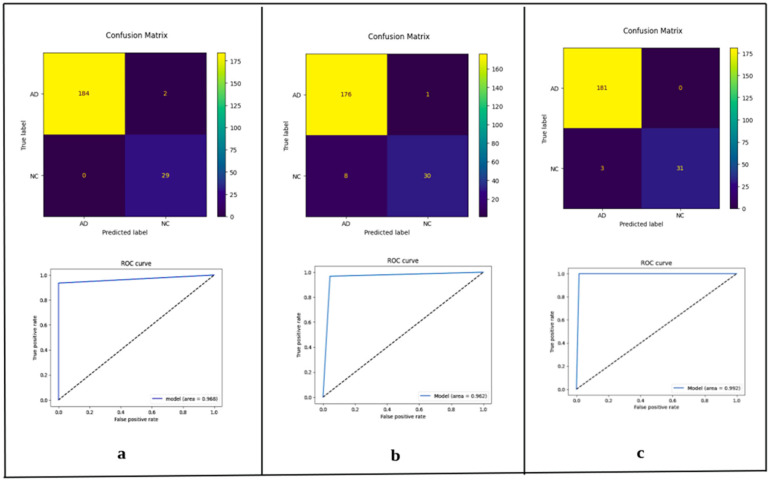
**Confusion Matrices and ROC Curves for ConvNeXt Variants on Coronal Plane Test Images.** Visual evaluation of classification performance on coronal MRI slices using the three ConvNeXt variants: (**a**) ConvNeXt-Tiny, (**b**) ConvNeXt-Small, and (**c**) ConvNeXt-Base. The top row depicts confusion matrices, where yellow cells indicate correct classifications and darker cells indicate misclassifications. The bottom row presents the corresponding Receiver Operating Characteristic (ROC) curves, with Area Under the Curve (AUC) values of 0.968, 0.962, and 0.992 for Tiny, Small, and Base, respectively. The results illustrate the high discriminative power of ConvNeXt models on coronal images, particularly the Base variant, which demonstrates near-perfect separation between Alzheimer’s disease (AD) and normal control (NC) classes.

**Figure 5:**
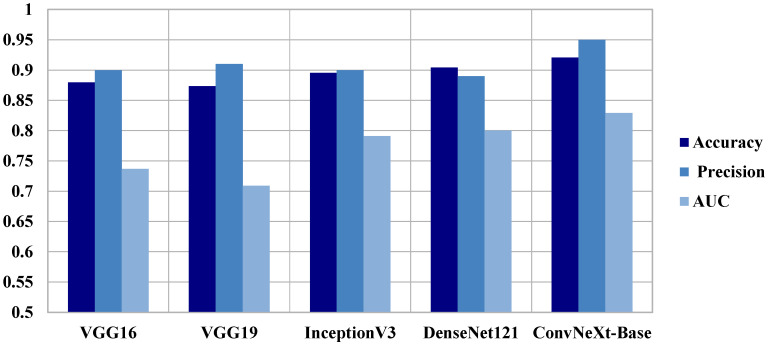
**Comparative Performance of ConvNeXt Versus Pre-trained Models Across Axial Plane Images.** Bar plot comparing the classification performance of ConvNeXt-Base against standard pre-trained convolutional models (VGG16, VGG19, InceptionV3, and DenseNet121) on axial MRI slices from the AlzaSet dataset. Metrics displayed include Accuracy, Precision, and Area Under the Curve (AUC). ConvNeXt-Base outperforms all benchmark models across all three evaluation metrics, highlighting its superior capacity for learning discriminative features relevant to Alzheimer’s pathology in axial views.

**Figure 6:**
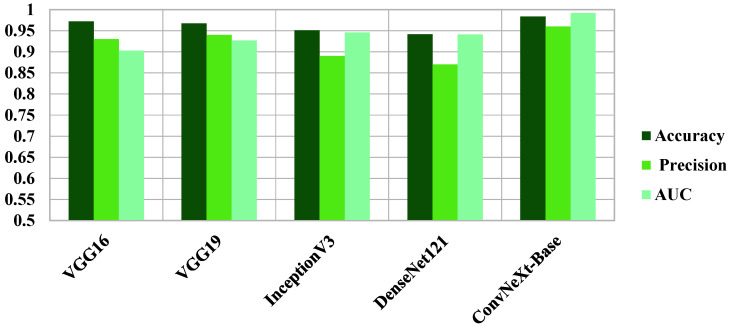
**Comparative Performance of ConvNeXt versus Pre-trained Models Across Coronal Plane Images.** Bar chart illustrating the performance comparison of ConvNeXt-Base against four widely adopted pre-trained CNN models, VGG16, VGG19, InceptionV3, and DenseNet121, on coronal MRI slices from the AlzaSet dataset. Evaluation metrics include Accuracy (dark green), Precision (medium green), and Area Under the ROC Curve (AUC; light green). ConvNeXt-Base demonstrates superior performance across all three metrics, particularly in AUC, underscoring the enhanced discriminatory power of coronal slices and the architectural advantage of ConvNeXt for capturing Alzheimer-related features in medial temporal lobe structures.

**Figure 7:**
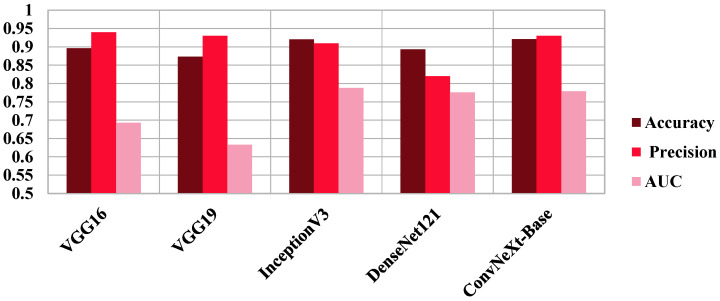
**Comparative Performance of ConvNeXt-Base Versus Pretrained CNN Models on Sagittal MRI Plane Images.** Performance comparison of ConvNeXt-Base against VGG16, VGG19, InceptionV3, and DenseNet121 on sagittal MRI slices from the AlzaSet dataset. ConvNeXt-Base achieved the highest accuracy (92.1%) and precision (94%), with strong AUC performance (0.779), highlighting its superior ability to detect Alzheimer’s disease in lateral brain views despite anatomical limitations.

To place our results in context, we compared ConvNeXt-Base with four well-known CNN architectures, VGG16, VGG19, InceptionV3, and DenseNet121, each with the same preprocessing and hyperparameter training. ConvNeXt-Base outperformed these established models in all the anatomical planes. In axial view, ConvNeXt-Base performed better than DenseNet121 by a good margin in both AUC (0.829 vs. 0.8002) and F1-score (0.870 vs. 0.830). In coronal view, ConvNeXt-Base recorded the highest AUC (0.992) and F1-score (0.970) among all models tested. In sagittal plane, ConvNeXt-Base also performed best in the majority of the metrics, demonstrating how it is robust across orientations. The superiority of ConvNeXt models is also better highlighted in Figure 4, Figure 5, Figure 6 and Figure 7, comparing performance on key classification metrics, and demonstrating clearly their discriminability and generalizability.

Confusion matrix plots and ROC curves (Figure 4) also strongly support the stability of the ConvNeXt-Base model. On the coronal test set, it had low misclassification and virtually perfect class separation, again strongly supporting the conclusion that architectural depth as well as anatomical orientation both have key roles to play in enhancing diagnostic accuracy.

## 4. Discussion

### 4.1. Interpretation of Findings and Architectural Insights

Our results solidly establish the effectiveness of ConvNeXt models for AD prediction from clinical images, particularly after training on high-quality datasets such as AlzaSet. ConvNeXt not only achieves better classification performance relative to traditional CNNs but also offers deployment advantages in practice with its modularity, efficiency, and interoperability with modern training schemes.

The ConvNeXt-Base configuration outperformed both legacy CNNs and smaller ConvNeXt variants, indicating that deeper, properly regularized representations are required to capture the subtle cortical and hippocampal atrophy patterns characteristic of Alzheimer’s pathology. When benchmarked against prior ConvNeXt-based frameworks for AD classification [25,26,38,39], our model achieved higher diagnostic accuracy (98.37%) while maintaining a streamlined, fully convolutional 2D design. Previous implementations using 3D or attention-augmented ConvNeXt architectures typically reported accuracies between 92% and 95%, often at substantially greater computational cost. This improvement highlights how optimized 2D orientation-specific training and dataset fidelity together drive superior performance with greater computational efficiency.

Furthermore, our plane-wise analysis revealed that coronal slices consistently yielded the maximum diagnostic accuracy in all models, supporting previous radiological evidence emphasizing medial temporal lobe visualization as key. Collectively, these findings demonstrate that a carefully optimized 2D ConvNeXt pipeline can achieve state-of-the-art accuracy while remaining lightweight and clinically scalable.

### 4.2. Integration with Prior Literature

These findings are also supported when compared with more recent work by Techa et al., where they built an ensemble structure for AD classification using ConvNeXt on a Kaggle dataset [38]. While their model achieved high accuracy (92.2%) using stacking classifiers, their pipeline introduced an extra layer of complexity by combining multiple machine learning classifiers (i.e., SVM, MLP, Decision Trees) post-feature extraction. Conversely, our effort utilized an end-to-end deep learning system, streamlining the pipeline and reducing inference overhead. Our model’s emphasis on orientation-specific evaluation (axial, coronal, sagittal) and use of an expert-annotated clinical dataset further distinguishes our approach both methodologically and in terms of clinical relevance.

In parallel, Li et al. applied a ConvNeXt-based deep learning framework to Alzheimer’s MRI classification, achieving similarly strong performance on open datasets and further underscoring the rapid convergence of the field on convolutional and transformer-inspired architectures [39]. Our work builds on these developments by introducing AlzaSet as a rigorously curated dataset and by systematically benchmarking across anatomical planes, thereby enhancing reproducibility and translational relevance. In addition, a recent study by Moscoso et al. (2025) in JAMA highlights the prognostic value of tau PET imaging, demonstrating that individuals who are both Aβ- and tau-positive have a significantly elevated risk of clinical conversion from preclinical to MCI or dementia [40]. This favors the usefulness of multimodal approaches, such as combining deep learning on structural MRI with PET-based biomarkers, to stage AD pathology earlier and more precisely. Such biomarker stratification incorporated into model training pipelines can enhance prognostic significance and clinical usefulness.

Another unique feature of the current work is in the target classification task. While Techa et al. addressed multi-class classification across different AD stages (non-demented, very mild, mild, moderate), our methodology aimed at binary classification (AD vs. NC), which is more clinically pertinent in the immediate sense for early screening and detection. In addition, AlzaSet’s dataset, developed in cooperation with clinical site’s neuroradiologists, provides higher consistency and diagnostic validity over the publicly available, unevenly annotated Kaggle dataset. These differences highlight the translational potential of our model.

### 4.3. Comparative Positioning Relative to Existing ConvNeXt-Based AD Classifiers

Our results position the proposed ConvNeXt-Base model at or above current state-of-the-art performance for MRI-based AD classification. Prior ConvNeXt-derived approaches have typically reported accuracies in the 92–95% range using either 3D convolutional backbones with attention modules or ensemble/stacked classifiers trained on public datasets [25,26,38,39]. For example, Hu et al. reported 94.23% accuracy using a 3D SE-augmented ConvNeXt architecture on Alzheimer’s MRI [25], and Jin et al. achieved 95.3% accuracy using a coordinate-attention ConvNeXt variant compared against ResNet50 and Swin Transformer [26]. Techa et al. achieved 92.2% using ConvNeXt features with an ensemble of downstream machine learning classifiers [38]. Li et al. similarly demonstrated strong ConvNeXt performance on open datasets [39].

In contrast, our ConvNeXt-Base model achieved 98.37% accuracy with an AUC of 0.992 on coronal slices from the clinically curated AlzaSet dataset, while retaining an end-to-end 2D convolutional design without stacking external classifiers or relying on attention-based 3D extensions. This indeed suggests that both: (i) orientation-specific optimization of coronal slices targeting medial temporal lobe anatomy and (ii) expert-labeled clinically acquired data are sufficient for performance competitive with or even surpassing that of more complex pipelines. Table 13 summarizes this comparison.

### 4.4. Clinical Implications and Future Directions

The clinical applicability of our results is significant. Notably, the ConvNeXt-Base model demonstrates classification accuracy on par with that of expert radiologists for binary diagnosis tasks, particularly for the coronal plane. In addition, model variants, Tiny and Base, offer computational cost vs. accuracy trade-offs for flexible deployment over a broad range of clinical settings. Compared to transformer-based or ensemble-based methods, ConvNeXt’s purely convolutional architecture maintains spatial locality, enabling easier application with post hoc interpretability techniques like Grad-CAM.

Furthermore, while we did not yet apply formal interpretability techniques (e.g., Grad-CAM) to spatially localize activation maps, the model’s superior performance on coronal slices strongly implicates disease-relevant changes in the medial temporal lobe. Specifically, ConvNeXt-Base could have learned to recognize early patterns of atrophy in the hippocampus, entorhinal cortex, and parahippocampal gyrus, regions frequently involved in prodromal and mild Alzheimer’s disease. These regions exhibit volume loss, thinning of the cortex, and architectural distortion on T1-weighted MRI and are optimally viewed in coronal planes. The use of SeparableConv2D and GlobalAveragePooling also enabled the model to compact localized structural abnormalities into informative features, which optimized its capability for classifying early disease stages. Subsequent studies using interpretability tools will allow a more precise ascription of these spatial features, possibly leading to earlier diagnostic triaging in the clinic.

### 4.5. Limitations

Yet, this study is not without limitations. Our database, although well-annotated, is from a single geographic site and hence cannot be expected to generalize to populations with varied demographics, or diverse imaging protocols. Another important limitation concerns demographic and sampling imbalance within the AlzaSet cohort. The AD group (70.1 ± 11 years) was significantly older than the NC group (57.7 ± 18 years), introducing a potential confounder given that normal aging is itself associated with cortical thinning and hippocampal atrophy. As a result, part of the model’s performance may reflect age-related rather than disease-specific structural changes. In addition, the dataset exhibits subject-level class imbalances (63 AD vs. 16 NC participants). Although image-level augmentation of NC scans helped to numerically balance training samples, it does not address the unequal number of subjects and may bias model learning towards the AD class. Moreover, we employed only T1-weighted MRI data; multimodal imaging inputs such as PET, diffusion-weighted MRI, or resting-state fMRI might serve to enhance model discriminability. Lastly, the binary nature of our classification task limits applicability to staging or longitudinal prediction of disease progression.

In addition, Because augmentation only expands images and not participants, it cannot correct subject-level skew; thus, classifier thresholds and apparent gains in sensitivity may overestimate performance in clinically balanced populations. Future work will focus on the recruitment of additional NC participants and external multi-site validation to equilibrate the class distribution at the subject level.

A further limitation relates to the timing of image acquisition. All MRIs used for model training were obtained after clinical diagnosis of Alzheimer’s disease and therefore represent relatively advanced disease stages already identified by treating physicians. While the model performs well in differentiating AD from normal controls under these conditions, its greatest clinical utility would be in detecting disease before clinical onset. Future studies should train and validate models on longitudinal cohorts that include individuals with mild cognitive impairment or early cognitive decline, ideally supplemented with neuropsychological testing, fluid biomarkers, or PET imaging for added specificity. In addition, higher-resolution characterization of the imaging features driving classification through activation mapping or voxel-wise morphometry will be required to precisely identify the earliest radiographic markers of disease that could inform preemptive diagnosis and treatment.

Additional future directions include expanding the dataset by including images from multiple institutions and integrating multi-modal neuroimaging data. Addition of attention-based architectures or hybrid CNN-transformer architectures could also provide increases in performance. Finally, leveraging self-supervised pretraining over large, unlabeled neuroimaging datasets could reduce data sparsity and further enhance generalizability. We have made our data publicly available in Supplementary Materials for further analysis by the scientific community.

Notably in the context of broader applicability, parallel advances beyond neurology, such as ensemble learning of pathology foundation models for precision oncology and safety-centric evaluation frameworks for clinical AI (CREOLA), show that combining complementary models with layered, stakeholder-informed monitoring improves robustness, generalizability, and clinician trust; we will incorporate these principles into future iterations of our AD pipeline [41,42].

## 5. Conclusions

Overall, this paper presents an efficient, scalable approach to automating Alzheimer’s disease diagnosis on 2D MRI images with ConvNeXt-based deep learning [43]. The presentation of the AlzaSet dataset, exploration of anatomical orientation, and comparative benchmarking against baseline CNNs all contribute to highlighting the ConvNeXt-Base architecture as a potentially optimal solution for clinical decision support in AD diagnostics. In contrast to ensemble-based pipelines, our minimalistic architecture maintains accuracy while facilitating model interpretability and efficiency. Following further validation and scale-up, this platform can assist in real-time clinical deployment, especially in resource-constrained environments, enabling earlier and more equitable diagnosis of Alzheimer’s disease worldwide.

## Figures and Tables

**Table 1:** Demographic information of AlzaSet.

Participant Status	Age	Gender (F/M)%
**AD**	70.1 ± 11	37/63
**NC**	57.7 ± 18	54/46

**Table 2:** The exact counts of images for each category of AlzaSet.

Participants Status	Axial	Coronal	Sagittal	Total
**AD**	4876	1801	4053	10,730
**NC**	1283	283	651	2217
**Total**	6159	2084	4704	12,947

**Table 3:** Distribution of split dataset.

Axial	Train	Validation	Test	Coronal	Train	Validation	Test	Sagittal	Train	Validation	Test
**AD**	3459	954	463	**AD**	1247	449	184	**AD**	2823	826	404
**NC**	913	240	130	**NC**	184	66	32	**NC**	408	160	83

**Table 4:** Distribution of AlzaSet after data balancing.

Axial	Train	Validation	Test	Coronal	Train	Validation	Test	Sagittal	Train	Validation	Test
**AD**	3459	954	463	**AD**	1247	449	184	**AD**	2823	826	404
**NC**	**3648**	**931**	130	**NC**	**1267**	**443**	32	**NC**	**2253**	**776**	83

**Table 5:** Model Training Hyperparameters.

Hyperparameter	Value	Description
**Batch Size**	32	Chosen due to GPU memory constraints.
**Loss Function**	Categorical Cross-Entropy	Selected due to its ability to measure the dissimilarity between predicted probabilities and true class labels, thereby simplifying optimization and enhancing model performance.
**Epochs**	35	Selected through validation (tested epochs: 20, 35, 50, 100) with 35 demonstrating model convergence.
**Optimizer**	Adam	Based on its demonstrated advantages in both optimization efficiency and generalization performance.
**Learning Rate**	1 × 10^−4^	The tested range (1 × 10^−3^ to 1 × 10^−5^) and final selection 1 × 10^−4^.

**Table 6:** Confusion Matrix for Classification.

	Predicted Positives	Predicted Negatives
Actual Positives	tp	fn
Actual Negatives	fp	tn

**Table 7:** Test Set Performance Metrics for Axial Plane Images.

Axial	Accuracy	Loss	Precision Macro Avg	Recall Macro Avg	F1-Score Macro Avg	AUC
**ConvNeXt-Tiny**	0.9303	0.2590	0.9400	0.8500	0.8800	0.8470
**ConvNeXt-Small**	0.8877	0.3388	0.9200	0.7500	0.8000	0.7480
**ConvNeXt-Base**	0.9208	0.2176	0.9500	0.8300	0.8700	0.8290

**Table 8:** Test Set Performance Metrics for Coronal Plane Images.

Coronal	Accuracy	Loss	Precision Macro Avg	Recall Macro Avg	F1-Score Macro Avg	AUC
**ConvNeXt-Tiny**	0.9896	0.1015	0.9900	0.9700	0.9800	0.9680
**ConvNeXt-Small**	0.9579	0.1339	0.8900	0.9600	0.9200	0.9620
**ConvNeXt-Base**	0.9837	0.0924	0.9600	0.9900	0.9700	0.9920

**Table 9:** Test Set Performance Metrics for Sagittal Plane Images.

Sagittal	Accuracy	Loss	Precision Macro Avg	Recall Macro Avg	F1-Score Macro Avg	AUC
**ConvNeXt-Tiny**	0.9077	0.3011	0.9200	0.7400	0.8000	0.7430
**ConvNeXt-Small**	0.8754	0.3208	0.8000	0.6800	0.7200	0.6830
**ConvNeXt-Base**	0.9209	0.1791	0.9300	0.7800	0.8300	0.7790

**Table 10:** Performance of Pre-trained Networks on Axial Plane Images of AlzaSet.

Axial	Accuracy	Loss	Precision Macro Avg	Recall Macro Avg	F1-Score Macro Avg	AUC
**VGG16**	0.8799	0.2771	0.9000	0.7400	0.7800	0.7370
**VGG19**	0.8734	0.3564	0.9100	0.7100	0.7600	0.7090
**InceptionV3**	0.8954	0.2006	0.9000	0.7900	0.8300	0.7910
**DenseNet121**	0.9043	0.2606	0.8900	0.8000	0.8300	0.8002

**Table 11:** Performance of Pre-trained Networks on Coronal Plane Images of AlzaSet.

Coronal	Accuracy	Loss	Precision Macro Avg	Recall Macro Avg	F1-Score Macro Avg	AUC
**VGG16**	0.9722	0.1780	0.9800	0.9400	0.9000	0.9030
**VGG19**	0.9678	0.2133	0.9400	0.9300	0.9300	0.9270
**InceptionV3**	0.9513	0.2966	0.8900	0.9500	0.9100	0.9460
**DenseNet121**	0.9415	0.2910	0.8700	0.9400	0.9000	0.9410

**Table 12:** Performance of Pre-trained Networks on Sagittal Plane Images of AlzaSet.

Sagittal	Accuracy	Loss	Precision Macro Avg	Recall Macro Avg	F1-Score Macro Avg	AUC
**VGG16**	0.8963	0.2573	0.9400	0.6900	0.7500	0.6930
**VGG19**	0.8731	0.2616	0.9300	0.6300	0.6700	0.6330
**InceptionV3**	0.9204	0.2003	0.9100	0.7900	0.8300	0.7880
**DenseNet121**	0.8936	0.2874	0.8200	0.7800	0.7900	0.7760

**Table 13:** Comparison of ConvNeXt-based Alzheimer’s disease (AD) MRI classification models in the literature versus the present study.

Study/Model (Ref)	Dataset Source	Task/Classes	Architecture Style	Reported Accuracy	Notes
Hu et al., 2025 [25] (“3D-SEConvNeXt”)	Public AD MRI cohort (3D volumes)	AD vs. NC	3D ConvNeXt + Squeeze-and-Excitation attention	94.23%	High-capacity 3D model with SE attention; higher compute cost.
Jin et al., 2022 [26] (“CA-ConvNeXt”)	ADNI	Multi-stage AD classification/staging	ConvNeXt + coordinate attention; compared with ResNet50, Swin Transformer	95.3%	Uses attention augmentation and compares to transformer baselines.
Techa et al., 2022 [38]	Kaggle MRI dataset	Multiclass dementia staging	ConvNeXt feature extractor + stacked ML ensemble (SVM, MLP, etc.)	92.2%	Ensemble/post hoc classifiers; heterogeneous public data.
Li et al., 2019 [39]	Public/open MRI datasets	AD vs. NC	ConvNeXt-based deep model	“High performance” (~92–95% range as reported)	Reported strong ConvNeXt performance on open data.
This work (ConvNeXt-Base)	AlzaSet (expert-labeled clinical MRI)	AD vs. NC (binary)	End-to-end 2D ConvNeXt-Base on coronal slices	98.37%	AUC 0.992; orientation-specific coronal training targeting medial temporal lobe anatomy; computationally lightweight.

## Data Availability

The AlzaSet dataset used in this study is available in Zenodo under embargo at the following DOI: https://doi.org/10.5281/zenodo.15564968, accessed on 17 November 2025. The dataset may be obtained by researchers on reasonable request from the corresponding authors. The data consist of anonymized T1-weighted structural MRI scans from Alzheimer’s disease and cognitively normal subjects, carefully curated and labeled by expert neuroradiologists. All releases of the dataset are cited under the above DOI, which will link to the most recent version.

## Supplementary Materials

The following supporting information can be downloaded at: https://www.mdpi.com/article/10.3390/diagnostics15232997/s1, Table S1: Hardware specifications; Table S2: Python Libraries.
