# Supplementary material for: ConvNeXt-Driven Detection of Alzheimer’s Disease: A Benchmark Study on Expert-Annotated AlzaSet MRI Dataset Across Anatomical Planes
Source: Diagnostics (Basel). 2025 Nov 25;15(23):2997. doi: 10.3390/diagnostics15232997 (PMC12691013; doi:10.3390/diagnostics15232997)
Supplement: Supplementary file 1 [file diagnostics-15-02997-s001.zip › diagnostics-3927611-supplementary.pdf]

Supplementary Materials for "ConvNeXt for Alzheimer's Detection: Benchmarking Modern Architecture on the AlzaSet MRI Dataset".

This document supplements the main manuscript with:

- Additional Results: Complete per-plane performance metrics, confusion matrices, and ROC curves. (`Supplementary-Materials/TestSet-results')
- Hardware specifications: All experiments were conducted on Google Colab free and Google Colab pro (Jun-July2024, Order number : COL.3347-8355-8490-08315) with the following resourses. (Table S1)

Table S1: Hardware specifications

Component	Specification
CPU	Intel Xeon (2.2+ GHz, 2 cores)
GPU	Tesla T4 (16GB) |V100 (16GB)
RAM	12-52GB

- Table S2: Python Libraries

Library	Version
TensorFlow	2.12.1 | 2.16.1
Keras	3.3.3
NumPy	1.26.4
Matplotlib	3.8.4
Seaborn	0.13.2

- AlzaSet Dataset: The AlzaSet dataset will be made publicly available on Github and Kaggle upon manuscript acceptance. Full access can be requested via email at baserehmahdiyeh@gmail.com during the review process.
